# Different mechanisms for resistance to trastuzumab versus lapatinib in HER2- positive breast cancers -- role of estrogen receptor and HER2 reactivation

**DOI:** 10.1186/bcr3067

**Published:** 2011-11-28

**Authors:** Yen-Chao Wang, Gladys Morrison, Ryan Gillihan, Jun Guo, Robin M Ward, Xiaoyong Fu, Maria F Botero, Nuala A Healy, Susan G Hilsenbeck, Gail Lewis Phillips, Gary C Chamness, Mothaffar F Rimawi, C Kent Osborne, Rachel Schiff

**Affiliations:** 1 Lester and Sue Smith Breast Center, Baylor College of Medicine, Houston, TX, USA; 2 Translational Biology and Molecular Medicine Program, Baylor College of Medicine, Houston, TX, USA; 3 Dan L. Duncan Cancer Center, Baylor College of Medicine, Houston, TX, USA; 4 Margaret M. and Albert B. Alkek Department of Medicine, Baylor College of Medicine, Houston, TX, USA; 5 Department of Molecular and Cellular Biology, Baylor College of Medicine, Houston, TX, USA; 6 Genentech, Inc., South San Francisco, CA, USA; 7 Department of Surgery, National University of Ireland, Galway, Ireland

## Abstract

**Introduction:**

The human epidermal growth factor receptor 2 (HER2)-targeted therapies trastuzumab (T) and lapatinib (L) show high efficacy in patients with HER2-positive breast cancer, but resistance is prevalent. Here we investigate resistance mechanisms to each drug alone, or to their combination using a large panel of HER2-positive cell lines made resistant to these drugs.

**Methods:**

Response to L + T treatment was characterized in a panel of 13 HER2-positive cell lines to identify lines that were *de novo *resistant. Acquired resistant lines were then established by long-term exposure to increasing drug concentrations. Levels and activity of HER2 and estrogen receptor (ER) pathways were determined by qRT-PCR, immunohistochemistry, and immunoblotting assays. Cell growth, proliferation, and apoptosis in parental cells and resistant derivatives were assessed in response to inhibition of HER or ER pathways, either pharmacologically (L, T, L + T, or fulvestrant) or by using siRNAs. Efficacy of combined endocrine and anti-HER2 therapies was studied *in vivo *using UACC-812 xenografts.

**Results:**

ER or its downstream products increased in four out of the five ER+/HER2+ lines, and was evident in one of the two intrinsically resistant lines. In UACC-812 and BT474 parental and resistant derivatives, HER2 inhibition by T reactivated HER network activity to promote resistance. T-resistant lines remained sensitive to HER2 inhibition by either L or HER2 siRNA. With more complete HER2 blockade, resistance to L-containing regimens required the activation of a redundant survival pathway, ER, which was up-regulated and promoted survival via various Bcl2 family members. These L- and L + T-resistant lines were responsive to fulvestrant and to ER siRNA. However, after prolonged treatment with L, but not L + T, BT474 cells switched from depending on ER as a survival pathway, to relying again on the HER network (increased HER2, HER3, and receptor ligands) to overcome L's effects. The combination of endocrine and L + T HER2-targeted therapies achieved complete tumor regression and prevented development of resistance in UACC-812 xenografts.

**Conclusions:**

Combined L + T treatment provides a more complete and stable inhibition of the HER network. With sustained HER2 inhibition, ER functions as a key escape/survival pathway in ER-positive/HER2-positive cells. Complete blockade of the HER network, together with ER inhibition, may provide optimal therapy in selected patients.

## Introduction

The human epidermal growth factor receptor 2 (HER2, ErbB2, or HER2/neu) is a member of the HER receptor tyrosine kinase (RTK) family, which includes three other members: epidermal growth factor receptor (EGFR or HER1), HER3, and HER4. Homo- and hetero-dimerization of ligand-bound HER receptors results in activation of multiple pathways, including the p44/42 mitogen-activated protein kinase (MAPK) and phosphatidylinositol 3-kinase (PI3K) pathways, which regulate cell proliferation and apoptosis [1-3]. HER2, the preferred heterodimerization partner of the other HER receptors, does not have a ligand and is activated by overexpression and homodimerization, or by ligand-mediated stimulation of another HER receptor through heterodimerization. Approximately 20% of human breast cancers are HER2-amplified, and overexpression correlates with aggressive tumor behavior and poor patient outcome [4].

To date, two distinct HER2-targeting agents, trastuzumab (T) and lapatinib (L), have been FDA-approved, and both have proven efficacy in the clinical setting [5-8]. Trastuzumab is a humanized monoclonal antibody that binds to the extracellular domain of HER2, disrupting HER signaling and inducing antibody-dependent cell-mediated cytotoxicity (ADCC) [9,10]. Lapatinib, a small-molecule EGFR/HER2 dual tyrosine kinase inhibitor (TKI), antagonizes the kinase activity of these receptors, inhibiting phosphorylation of their substrates and downstream signaling [11,12]. Despite their proven clinical benefit, *de novo *and acquired resistance to both L and T is common [13,14].

The HER signaling system has been described as a complex, robust, and redundant biological network, modulated by positive and negative feedback circuits [2]. These features, which protect the system from various perturbations, can also play a key role in resistance to drugs targeting this pathway. As such, multiple escape mechanisms to circumvent inhibition of the HER system have been reported to cause resistance [15,16], including compensatory activation of the HER network [17-19] or activation of other redundant survival pathways in the cell [20,21]. Therefore, multi-targeted therapies might be the optimal approach to prevent resistance in some patients.

Multiple levels of crosstalk between estrogen receptor (ER) and HER2 have been identified [20,21]. Our laboratory has previously shown that HER2 overexpression contributes to *de novo *and acquired resistance in various endocrine therapies [22,23]. Similarly, in the clinical setting, gene amplification of HER2 is associated with resistance to endocrine therapy [24-26]. Conversely, anecdotal observations from the clinic showed up-regulation of ER following treatment with trastuzumab in several patients with HER2-positive tumors [27-29]. Likewise, a retrospective study suggested a greater benefit of lapatinib in those patients with HER2-amplified tumors that are ER- and PR-negative, compared with hormone receptor positive patients [30]. An ER-positive/HER2-positive breast cancer cell line, BT474, has been reported to acquire resistance to lapatinib *in vitro *by up-regulating ER [20,21]. However, it is not yet fully established if this up-regulation of ER expression and/or activity can function as an escape mechanism to cause resistance to HER2 targeted therapy in other cell lines or in human breast cancer.

We and others previously hypothesized that a common mechanism of resistance to single agent anti-HER2 therapy is the incomplete blockade of the HER pathway and its multiple potential homo- and heterodimer pairs. We then reported that combination regimens including L + T were superior to single agent therapy and were capable of eradicating most HER2-positive xenografts *in vivo *[24,31]. However, some tumors still developed acquired resistance. In addition, we also showed that optimal antitumor effect in one cell line, MCF7-HER2, required endocrine therapy to block ER.

To further study the mechanisms of resistance to HER2-targeted therapies, we developed a panel of over 10 different HER2-positive human breast cancer cell lines *de novo *or acquired resistant to T, L, or L + T. We find that while *de novo *and acquired resistance to T is associated with reactivation of the HER2 pathway, resistance to L or L + T is due to alternative signaling through the ER pathway, providing clues to strategies to improve HER2-targeted therapies in the clinic.

## Materials and methods

### Cell lines and reagents

The human breast cancer cell line BT474 was obtained from AstraZeneca (Cheshire, UK) [24]. UACC-812, AU-565, and HCC-1569 cell lines were purchased from the American Type Culture Collection (Manassas, VA, USA). MDA-MB-361, MDA-MB-453, HCC-1954, ZR75-30, SKBR-3, and HCC-202 cell lines were obtained from Dr. Joe Gray (Berkeley Lab, Berkeley, CA, USA) [32]. SUM-190 and SUM-225 cells were obtained from Dr. Stephen Ethier (Wayne State University, Detroit, MI, USA). MCF7-HER2 cells were established as previously described [33]. BT474, UACC-812, MDA-MB-361, and MDA-MB-453 cell lines were maintained in Dulbecco's modified Eagle medium (DMEM) supplemented with 10% heat-inactivated fetal bovine serum (FBS) and 1% penicillin-streptomycin-glutamine (PSG). AU-565, HCC-1569, HCC-1954, ZR75-30, and HCC-202 cells were cultured in RPMI 1640 with 10% heat-inactivated FBS and 1% PSG. SKBR3 cells were grown in McCoy's 5A with 10% heat-inactivated FBS and 1% PSG. SUM-190 cells were maintained in Ham's F12 media with 5 μg/ml insulin, 1 μg/ml hydrocortisone, 5 mM ethanolamine, 10 mM HEPES, 5 μg/ml transferrin, 10 nM triiodothyronine, 50 nM sodium selenite, and 0.5 g/l bovine serum albumin (BSA). SUM-225 cells were grown in Ham's F12 media with 5% heat-inactivated FBS, 1% PSG, 5 μg/ml insulin, and 1 μg/ml hydrocortisone. Cell lines resistant (R) to HER2-targeted therapy were generated by long term culture of the cells in their original media with increasing concentrations of trastuzumab (1 to 50 μg/ml), lapatinib (0.1 to 1 μM), or both. For cells showing no growth inhibition, the treatment duration was at least three months, while responsive cells were cultured with their respective treatments until growth resumed. The time to the development of resistant growth varied from 3 to 12 months.

Trastuzumab (Herceptin) was acquired from Genentech (San Francisco, CA, USA) and dissolved in sterile distilled water. Lapatinib (Tykerb) was obtained from GlaxoSmithKline (US headquarters in Research Triangle Park, NC, USA) and prepared with dimethyl sulfoxide (DMSO). Fulvestrant (Faslodex) was obtained from AstraZeneca and prepared with ethanol.

### Cell growth assay

A total of 5,000 cells/well of the parental or resistant cell lines, cultured with their individual treatments, were plated in 96-well plates 24 hours before beginning respective additional treatments, which consisted of 10 μg/ml trastuzumab, 1 μM lapatinib, the combination of trastuzumab with lapatinib, or 10^-7 ^M fulvestrant. Cell growth was assessed at different time points (zero, three, six, and nine days). Cell cultures were fixed with 4% glutaraldehyde and stained with 0.05% methylene blue. The dye was subsequently extracted with 3% HCl and absorbance measured at 655 nm. Growth fold change was determined by ((O.D. 655 nm at six days/O.D. 655 nm at zero days) Treatment)/((O.D. 655 nm at six days/O.D. 655 nm at zero days) Control). Growth curve and growth fold change experiments were executed in quadruplicate.

### Immunohistochemistry (IHC)

Cells were fixed in 10% neutral buffered formalin prior to processing and paraffin embedding. Blocks were then organized into a 3-mm core tissue array and IHC was performed on 3-micron sections from these arrays [24]. Briefly, after deparaffinization, sections were subjected to epitope retrieval in tris-HCl buffer (pH 9.0) and then blocked in 3% hydrogen peroxide for 10 minutes. Slides were incubated with primary antibody to ER (Vector Labs, Burlingame, CA, USA), PR (Dako Cytomation, Carpinteria, CA, USA), or phospho-HER2-Tyr877 (Cell Signaling Technology, Beverly, MA, USA), for one hour. Immunodetection was performed with the EnVision+ System (Dako Cytomation, Carpinteria, CA, USA).

### Immunoblotting assay

Cells were lysed in buffer consisting of 10% Triton X100, 50 mM Hepes (pH 7.4), 150 mM NaCl, 1.5 mM MgCl_2_, 1 mM EGTA, 100 mM NaF, 10 mM NaPPi, 10% glycerol, 1 mM Na_3_VO_4_, and 1X protease inhibitor cocktail (Roche Molecular Biochemicals, Indianapolis, IN, USA). Protein lysates were collected and microcentrifuged at 14,000 *g *for 10 minutes at 4°C. Cell supernatants were aliquoted and stored at -80°C. Protein concentration was measured using the Bio-Rad Protein Assay kit (Bio-Rad Laboratories, Hercules, CA, USA) according to the manufacturer's directions. Equivalent amounts of protein (25 μg) from each sample were separated under denaturing conditions by electrophoresis on polyacrylamide gels containing sodium dodecyl sulfate (SDS-PAGE) and transferred by electroblotting onto nitrocellulose membranes (Invitrogen, Carlsbad, CA, USA). The blots were first stained with Ponceau S to confirm uniform loading and transfer, followed by immunoblotting with the specific primary antibodies according to the manufacturer's instructions. Briefly, blots were blocked with appropriate blocking buffer and then reacted at 4°C with primary antibodies at dilutions as per the manufacturer's directions overnight. Primary antibodies were: phospho-EGFR-Tyr1173 (Epitomics, Burlingame, CA, USA), EGFR, phospho-HER2- Tyr877, phospho-HER2-Tyr1221, HER2, phospho-HER3-Tyr1289, phospho-AKT-Thr308, phospho-AKT-Ser374, AKT, phospho-p44/42 MAPK- Thr202/Tyr204, p44/42 MAPK, β-actin, insulin-like growth factor-I receptor (IGF1R), cleaved PARP, caveolin-1 (Cav-1), Bik (all from Cell Signaling Technology), phospho-HER2-Tyr1248, HER3 (from Millipore, Billerica, MA, USA), ERα (Lab Vision, Fremont, CA, USA), progesterone receptor (PR), Cyclin-D1, and Bcl2 (from Santa Cruz Biotechnology, Santa Cruz, CA, USA). Blots were then incubated with a horseradish peroxidase-linked or a fluorescently-labeled secondary antibody for one hour, after which the labeled proteins were visualized by chemiluminescence or by the Odyssey Infrared Imaging System (LI-COR Biosciences, Inc., Lincoln, NE, USA). Gels were produced at least three independent times. For HER quantitation, protein levels of three independent samples from each resistant cell line were quantified with the Odyssey Infrared Imaging System and normalized to β-actin (protein levels/actin levels).

### Quantitative reverse transcription-polymerase chain reaction (qRT-PCR)

Total RNA was extracted using the RNeasy Mini kit (Qiagen, Valencia, CA, USA) according to the manufacturer's directions. For ER and PR analysis, the cDNA of each sample was generated by Superscript II reverse transcriptase and random hexamers (Invitrogen). Real time quantitative PCR (qRT-PCR) was performed using SYBR Green PCR Master Mix (ABI, Carlsbad, CA, USA), with human β-actin acting as an endogenous control. For analysis of HER ligands and receptors, gene expression was quantified using 100 ng of total RNA and Taqman One-Step Universal Master Mix in each qRT-PCR reaction, as described previously [19]. Normalization of EGFR family receptor and ligand gene expression was performed using the house-keeping gene *HP1BP3 *(heterochromatin protein 1, binding protein 3). All qRT-PCR reactions were performed in triplicate in a standard 96-well plate format with the ABI 7500 Real-Time qPCR System. Fold changes in mRNA expression were determined by the 2-ΔΔCt method. Target primer and probe sequences are available in supplemental material (Additional file 1).

### Xenograft studies

UACC-812 (ER-positive/HER2 amplified) cells were maintained as described in the "Cell lines and reagents" section. Animal care was in accordance with institutional guidelines. UACC-812 (ER-positive/HER2 amplified) xenografts were established in ovariectomized five- to six-week-old athymic mice (Harlan Sprague Dawley, Madison, WI, USA) supplemented with estrogen pellets by inoculating 5 × 10^6 ^cells subcutaneously as described previously [24]. When tumors reached the size of 150 to 200 mm^3 ^(two to four weeks), mice bearing the UACC-812 xenografts were randomly allocated to eight treatment groups, including continued estrogen (E2), E2 plus trastuzumab, E2 plus lapatinib, E2 plus the combination regimen (L + T), estrogen deprivation alone (ED) by removal of the estrogen pellets, ED plus trastuzumab, ED plus lapatinib, and ED plus the combination regimen. Each treatment group contained a minimum of 12 mice. Tumor volumes were measured weekly as previously described [24]. Each tumor analyzed was from a different mouse.

### siRNA transfection

Pooled small-interfering RNA (siRNA) oligos targeting EGFR, HER2, HER3, ERα, and nontargeting siRNA were purchased (Dharmacon, Lafayette, CO, USA). Cells were transfected with siRNA by reverse transfection per the manufacturers' directions. Briefly, 5,000 cells/well were seeded into 96-well plates containing a pre-incubated mixture of pooled siRNA oligos at 50 nM final concentration and Lipofectamine RNAiMax (Invitrogen) diluted in Opti-MEM (Invitrogen). The appropriate cell-specific medium supplemented with the relevant, respective drugs was added 24 hours after transfection and the effect of siRNA was determined after an additional 48 hours. For parallel protein expression analysis, 2 Χ 10^5 ^cells/well were plated into six-well plates and subjected to the transfection protocol as above.

### *In vitro *cell proliferation assay and apoptosis assay

The cell proliferation assay was performed using the Click-iT EdU (5-ethynyl-2'-deoxyuridine) Microplate Assay (Invitrogen) according to the manufacturer's directions. Following transfection with siRNA for 72 hours, cells were cultured with 10 μM EdU for 4 hours and the proliferation rate was analyzed by the Celigo Cytometer (Cyntellect, San Diego, CA, USA). Change in percent cell proliferation within parental and resistant derivatives was calculated as ((percentage of EdU-incorporating cells transfected with target siRNA/percentage of EdU-incorporating cells transfected with nontargeting siRNA) Χ 100). All measurements were performed in quadruplicate. Apoptosis assays were performed using the Annexin V-FITC Apoptosis Detection Kit (Abcam, Cambridge, MA, USA). Cells transfected with siRNA for 72 hours were incubated with Annexin V-FITC and DAPI for 30 minutes and apoptosis was analyzed by the Celigo Cytometer (Cyntellect, San Diego, CA, USA). Change in percent apoptosis was calculated as ((percentage of Annexin-V positive cells transfected with target siRNA/percentage of Annexin-V positive cells transfected with nontargeting siRNA) Χ 100). All measurements were performed in triplicate.

### Statistical analysis

Experiments assessing proliferation and apoptosis of various cell-lines under various treatment conditions were analyzed using one-way ANOVA. Data were log-transformed to stabilize variances. Differences between groups were determined by multiple comparisons using contrasts, and the Sidak method for *P*-value adjustment. Growth curve and growth fold change data *in vitro *were analyzed similarly. Error bars on plots represent +/- standard error (SE).

Xenograft tumor growth curves were constructed using the mean tumor volume at each time point with error bars representing the standard error of the mean. Animals that died of other causes prior to the first animal developing a resistant tumor were not included in the calculation of tumor growth curves. *P*-values for the xenograft studies were adjusted for multiple comparisons using the Hommel method to control for type I error when appropriate [34]. Progression of the tumor was defined as: tumor size more than zero and at least two consecutive measurements with ≧10% increments in tumor size. Time to progression (PFS) is the day of the measurement on which the tumor qualifies as a progression.

## Results

### Effect of combined lapatinib and trastuzumab (L+T) on a panel of HER2-positive breast cancer cell lines

**Figure 1:**
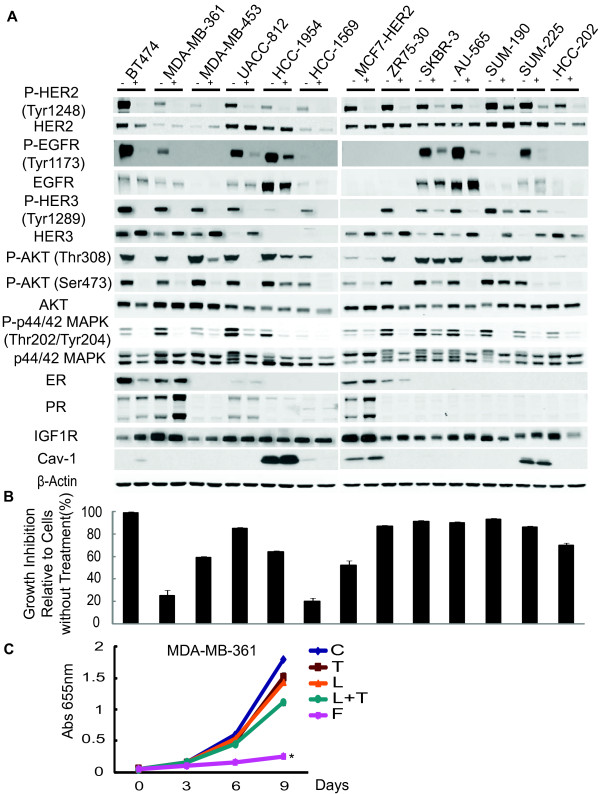
**HER2-overexpressing cell lines exhibit distinct responses when treated with potent anti-HER2 therapy**. **(A) **A panel of HER2-overexpressing breast cancer cell lines was treated with lapatinib (1 μM) plus trastuzumab (10 μg/ml) for 48 h and whole-cell extracts were analyzed by immunoblotting with indicated antibodies. **(B) **Combination therapy (trastuzumab plus lapatinib) growth response in the HER2-overexpressing breast cancer cell line panel. Growth inhibition was determined by methylene blue assay. Shown are the percent inhibitions of cells treated for six days normalized to non-treated cells. The experiment was performed in quadruplicate. Error bars on plots represent +/- standard error (SE). **(C) **Growth curves of *de novo *resistant MDA-MB-361 cells treated with different target therapies/regimens for nine days: trastuzumab (T) (10 μg/ml), lapatinib (L) (1 μM), trastuzumab plus lapatinib (L + T), or endocrine therapy, fulvestrant (F) (10^-7 ^M), untreated (C). Cell numbers were quantified by absorbance at 655 nm after staining with methylene blue. Conditions were repeated in quadruplicate. Significance between groups was determined by multiple comparisons using the Sidak method (*P < 0.0001, F versus C, T, L, or L + T).

We have previously shown in two HER2-positive breast cancer cell lines that the combination of trastuzumab (T) and lapatinib (L) more effectively inhibits HER downstream signaling and xenograft tumor growth than either monotherapy alone [31]. To investigate this potent combination in a broader representation of HER2-positive breast cancer subtypes, we used a panel of 13 different HER2-positive breast cancer cell lines with diverse genetic profiles and biological characteristics, representing both luminal and basal phenotypes [32,35,36]. Additional file 2 shows the cell lines and their general characteristics. Cells were treated with T (10 μg/ml) plus L (1 μM) for 48 hrs and inhibition of the HER pathway was evaluated by measuring phosphorylated EGFR, HER2, HER3, and key signal transduction mediators, including AKT and p44/42 MAPK (Figure 1A). All 13 cell lines showed significant inhibition of phosphorylated EGFR (Tyr1173), HER2 (Tyr1248), and HER3 (Tyr1289). The activity of downstream signaling mediators including phosphorylated AKT and p44/42 MAPK was also dramatically decreased in all except two lines, SKBR3 and SUM-190, which maintained comparable levels of phosphorylated AKT (Thr308) or showed slight reduction in phosphorylated AKT (Ser473) before and after treatment. Thus the combination regimen is highly effective in suppressing the HER pathway in most HER2-overexpressing models. Interestingly, the expression levels of total HER proteins, especially HER3, showed significant increases after the 48-hour treatment in 10 out of 13 models.

We also assessed changes in estrogen receptor (ER) level or its downstream gene products upon L + T treatment. Four out of the five ER-positive cell line models, BT474, MDA-MB-361, UACC-812, and MCF7-HER2, showed up-regulation of ER and/or one or more ER-regulated genes (PR, IGF1R, and Cav-1) following treatment, suggesting increased classical ER signaling activity.

The induction of ER activity or increased HER3 expression could potentially function as mechanisms of *de novo *resistance and, therefore, we investigated the effect of this regimen on tumor cell proliferation by analyzing growth inhibition after six days of treatment (Figure 1B). Eleven out of 13 lines showed substantial growth inhibition (> 50%) with L + T treatment, including MDAMB-453 and SUM-225 cell lines, in which HER2 is overexpressed but not gene-amplified. These results suggest that the up-regulation of HER receptor expression, most noticeably HER3, the incomplete inhibition of phosphorylated AKT, or the increased ER expression/signaling that occurred in several cell lines were insufficient to cause *de novo *resistance to short-term treatment (6 day) with L + T.

The HCC-1569 and MDA-MB-361 cell lines, however, demonstrated relative *de novo *resistance, as only modest growth inhibition was observed in response to L + T. The reduced sensitivity to L + T in HCC-1569 cells may be due to the overexpression of Cyclin E as previously described [32]. The MDA-MB-361 cell line showed marked up-regulation of ER and PR shortly after commencing treatment with L + T. Therefore, we asked whether ER might be the mechanism for *de novo *resistance in this model. We also investigated the effects of T and L, alone, in this model. While cell growth was only minimally inhibited by T, L, or the combination, it was significantly inhibited by fulvestrant (F) (*P *< 0.0001, F versus each treatment arm respectively) (Figure 1C), indicating that these cells are highly dependent on ER despite being amplified for HER2. These results suggest that some ER-positive/HER2-positive breast cancer cells might be primarily driven by ER and, thus, are intrinsically less sensitive to even potent anti-HER2 treatment.

### Characterization of cell lines with acquired resistance to T, L, and L+T

Since high ER activity can provide an escape pathway to reduce the efficacy of and cause *de novo *resistance to HER2-targeted therapies, we next asked whether up-regulated ER expression and/or activity might cause acquired resistance. The two cell lines (BT474 and UACC-812) that are amplified for HER2 and that showed up-regulated ER expression and/or activity after treatment with L + T were chosen for this set of experiments, with the parental lines demonstrating high (BT474) or very low (UACC-812) ER expression.

**Figure 2:**
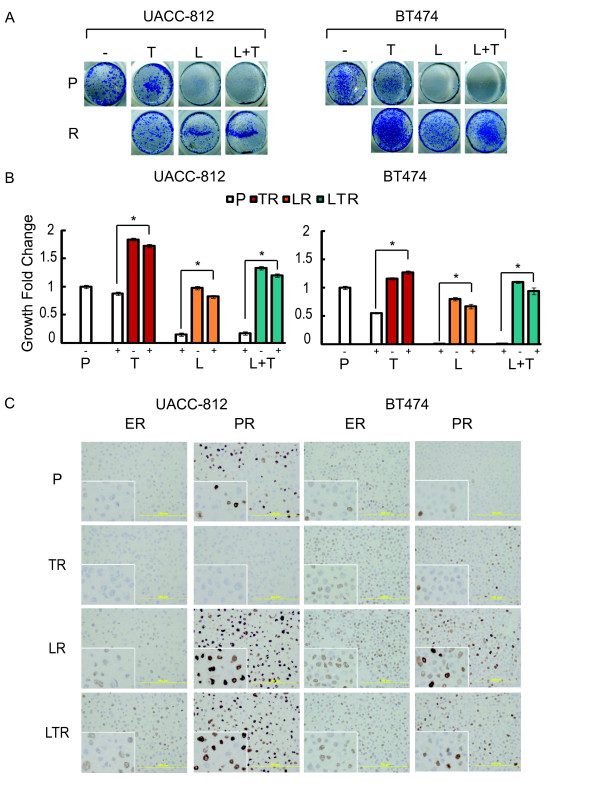
**Resistant cells show greater proliferation and exhibit changes in ER and PR expression**. **(A) **Cell proliferation assay of UACC-812 and BT474 parental and resistant (R) cells. Cells were treated with trastuzumab (T, 10 μg/ml), lapatinib (L, 1 μM), or trastuzumab plus lapatinib (L + T). After six days, viable cells were visualized by methylene blue staining and photographed. **(B) **Fold changes in cell growth of UACC-812 and BT474 parental and resistant cells with or without the respective anti-HER2 therapies, following six days of treatment. Cell numbers were quantified by absorbance at 655 nm and normalized against Day 0. Significance between groups was determined by multiple comparisons using the Sidak method (*P < 0.0001, P:T versus TR:T, P:L versus LR:L, or P:L + T versus LTR:L + T for both models). **(C) **Immunohistochemical detection of ER and PR in BT474 and UACC-812 parental and distinct resistant clones.

To characterize the response and resistance in these two models to different anti-HER2 therapies, parental cells (P) and resistant derivatives (R) were treated with T, L, or the combination regimen for six days (Figure 2A, B). Parental UACC-812 cells are *de novo *resistant to T (*P *= 0.2419, P: 0 versus T), but sensitive to L (*P *< 0.0001, P: 0 versus L) or the combination of L + T (*P *< 0.0001, P: 0 versus L + T). Parental BT474 cells showed sensitivity to all anti-HER2 therapies (*P *< 0.0001, P: 0 versus T, L, or L + T,), with L-containing regimens inhibiting growth more completely than T. In contrast, in the resistant derivatives there were no significant differences in cell growth in the presence or absence of the respective treatments. The cell lines resistant to T, L, and the combination showed significantly higher proliferation rates than parental cells in the presence of the respective treatments (*P *< 0.0001, P:T versus TR:T, P:L versus LR:L, or P:L + T versus LTR:L + T), suggesting that resistant derivatives resumed growth and, indeed, had acquired resistance to HER2-targeted therapies. Overall, the resistant cells with or without treatment grew at a rate similar to or faster than parental cells in the absence of treatment.

**Figure 3:**
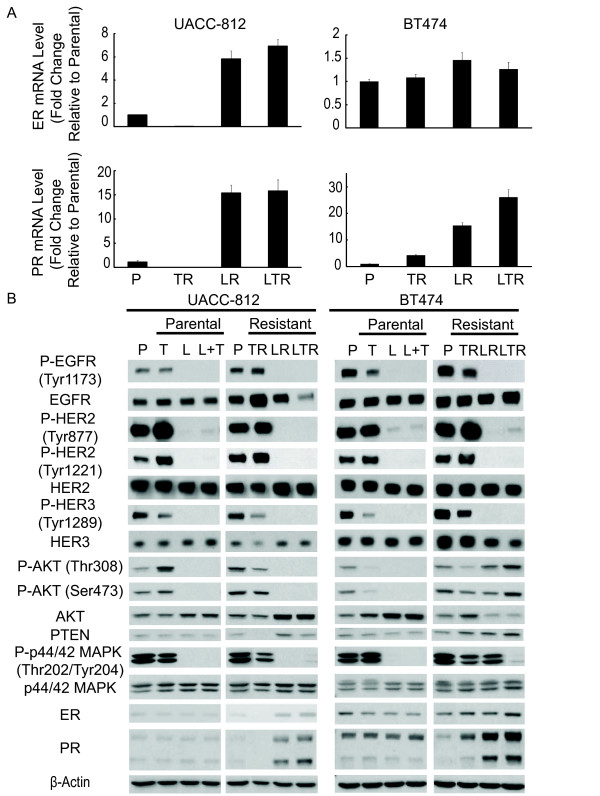
**Trastuzumab resistant cells maintain HER signaling. Lapatinib and combination resistant cells express up-regulated ER activity**. **(A) **qRT-PCR expression levels of ER and PR mRNA in UACC-812 and BT474 parental and distinct resistant clones. Data were normalized to parental cells. **(B) **UACC-812 and BT474 parental cells were treated with trastuzumab (10 μg/ml), lapatinib (1 μM), or trastuzumab plus lapatinib for five hours and harvested. Whole-cell extracts of these treatment groups and resistant derivatives were analyzed by Western blot with the indicated antibodies.

Immunohistochemistry (IHC) and qRT-PCR on UACC-812 and BT474 parental and resistant derivatives (Figures 2C and 3A) revealed that the low levels of ER mRNA and protein remained low/undetectable in TR cells but the more substantial PR protein level of parental UACC812 cells was completely lost in the TR cells. PR mRNA was low in both parental and TR UACC-812 cells. No substantial changes in ER or PR levels were observed in BT474 TR cells. In contrast to TR cells, LR and LTR derivatives of both UACC-812 and BT474 displayed a marked increase in ER and/or PR protein levels. PR mRNA levels were also markedly increased in both UACC-812 and BT474 LR and LTR cells. While ER mRNA also dramatically increased in UACC-812 LR and LTR cells, only a modest increase in expression was observed in BT474 parental and resistant derivatives. These results suggest that ER expression and/or classical transcriptional activity are correlated with acquired resistance to both L and the L + T combination in these HER2-positive breast cancer models.

We further determined the phosphorylation status of the HER receptors and their downstream mediators, AKT and p44/42-MAPK, in the parental and resistant derivatives (Figure 3B). To evaluate the primary response of the parental cell lines to anti-HER2 therapies, parental UACC-812 and BT474 cells were treated with T (10 μg/ml), L (1 μM), or the combination therapy for five hours. We found that T inhibited the phosphorylation of HER3 and partly inhibited phosphorylated EGFR in BT474 cells, while in UACC-812 cells reduced phosphorylated HER3 but not phosphorylated EGFR was observed. This observation is consistent with published reports which suggest a mechanism of action for trastuzumab involving disruption of ligand-independent HER2/HER3 signaling in HER2-positive cells [37]. Interestingly, while phosphorylated AKT was reduced by trastuzumab in BT474 cells, it was increased slightly in the UACC-812 line which is relatively *de novo *resistant to T. However, L and L + T markedly suppressed the entire HER pathway and the downstream MAPK and AKT kinases in both UACC-812 and BT474 cells. Collectively, these results suggest that L-containing regimens more effectively inhibit the HER signaling pathway than T.

Immunoblot analysis of the parental BT474 and resistant derivatives showed that cells resistant to T (TR) maintained or reactivated the HER signaling pathway (Figure 3B). However, cells resistant to L or L + T, in which the HER receptor layer is more completely inhibited, continued to show marked suppression of phosphorylated EGFR, HER2, and HER3. In contrast to TR cells, LR and LTR cells displayed high levels of PR. Despite a reduction in total AKT and reduced levels of phosphorylated EGFR, HER2, and HER3, LR and LTR, cells showed a slight increase in phosphorylated AKT. UACC-812 resistant cells behaved in a similar manner, where TR clones demonstrated enhanced HER signaling, while L and the L + T resistant derivatives showed enhanced ER activity in the wake of suppressed HER signaling. Of note, a decrease in PTEN expression level was observed in UACC-812 TR cells, but not in BT474 TR cells.

### Growth characterization of resistant cell lines with HER2 and ER targeted therapies reveals their differential role in resistance to trastuzumab versus lapatinib containing regimens

**Figure 4:**
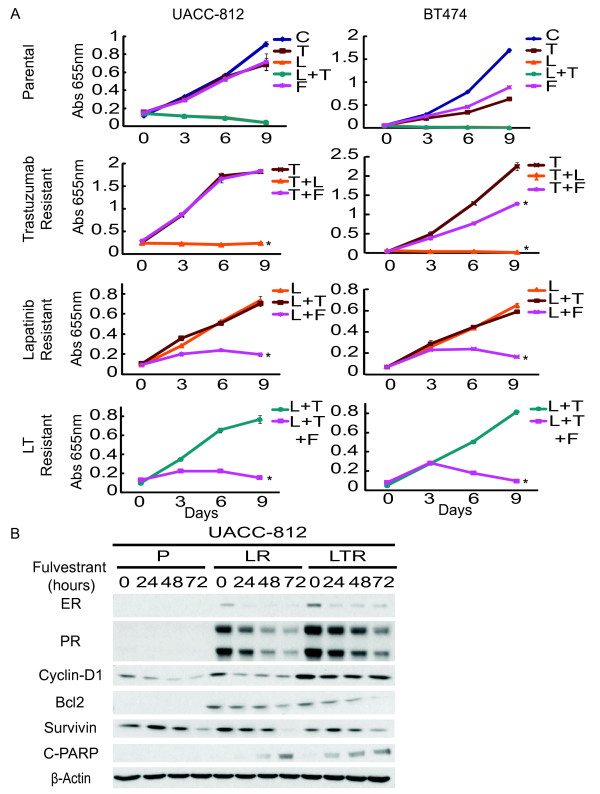
**Trastuzumab resistant cells remain sensitive to lapatinib. Fulvestrant inhibits lapatinib and combination resistant cell growth**. **(A) **Growth curves of UACC-812 and BT474 parental and resistant cells treated with different target therapies/regimens for nine days: trastuzumab (T) (10 μg/ml), lapatinib (L) (1 μM), trastuzumab plus lapatinib (L + T), or endocrine therapy, fulvestrant (F) (10^-7^M); media of parental cells (C). Cell numbers were quantified by absorbance at 655 nm after staining with methylene blue. Conditions were repeated in quadruplicate. Significance between groups was determined by multiple comparisons using the Sidak method (*P < 0.0001, UACC-812 TR:T versus TR:T + L, LR:L versus LR:L + F, or LTR:L + T versus LTR:L + T + F; BT474 TR:T versus TR:T + L, TR:T versus TR:T + F, LR:L versus LR:L + F, or LTR:L + T versus LTR:L + T + F). **(B) **UACC-812 parental, lapatinib resistant, and combination resistant cells were treated with fulvestrant for 24, 48, 72 h and whole-cell extracts were analyzed by Western blot with the indicated antibodies.

To investigate whether up-regulated HER and/or ER pathways are responsible for the proliferative and survival stimuli of the resistant derivatives, parental and resistant BT474 and UACC-812 lines were treated with T (10 μg/ml), L (1 μM), the combination regimen, or the anti-estrogen fulvestrant (F) (10^-7 ^M) (Figure 4A). Cell growth was followed over nine days. Consistent with their molecular profiling data, both BT474 TR and UACC-812 TR were still dependent on HER2 and, therefore, sensitive to L (*P *< 0.0001, TR + T versus TR + T + L for both models). UACC-812 TR showed no response to F (*P *= 0.7004, TR + T versus TR + T + F for UACC-812 TR), and BT474 TR sustained the same modest sensitivity to F as parental cells. In both models, LR derivatives were also resistant to T (*P *= 0.8901, LR + L versus LR + L + T for UACC812 LR; *P *= 0.0788, LR + L versus LR + L + T for BT474 LR). Conversely, however, LR and LTR cells, but not parental cells, were highly sensitive to anti-ER therapy with F. These results suggest that ER activity plays a minimal role, if any, in TR cells where the HER pathway remains the dominant driver of cell growth and where TR cells are inhibited by L. In contrast, up-regulated ER activity becomes the dominant driver in cells resistant to L and L + T.

**Figure 5:**
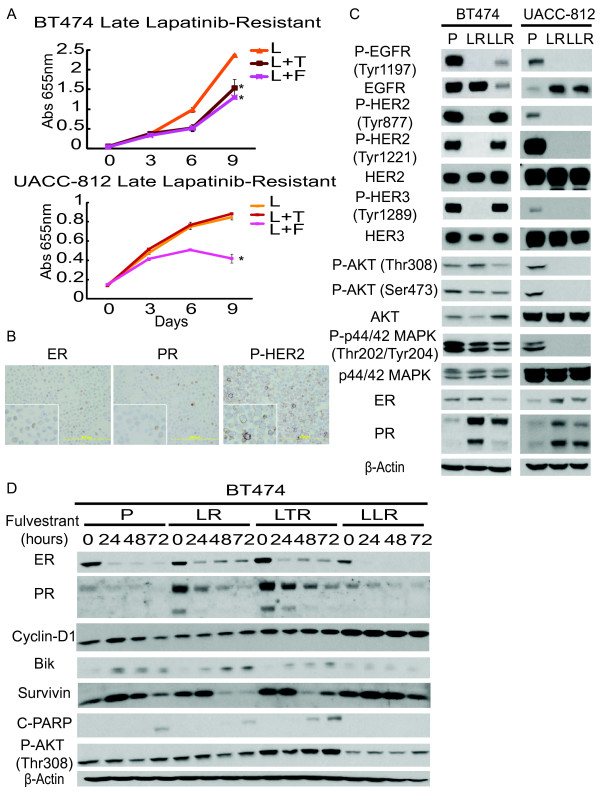
**BT474 lapatinib resistant cells with prolonged treatment reactivate HER receptor activity**. **(A) **Growth curves of UACC-812 and BT474 late stage lapatinib resistant cells (LLR) treated with different targeted therapies for nine days: trastuzumab (T) (10 μg/ml), lapatinib (L) (1 μM), trastuzumab plus lapatinib, or endocrine therapy, fulvestrant (F) (10^-7^M). Significance between groups was determined by multiple comparisons using the Sidak method (*P = 0.0008, BT474 LLR + L versus LLR + L + F, *P = 0.0044, BT474 LLR + L versus LLR + L + T; *P < 0.0001, UACC-812 LLR + L versus LLR + L + F). **(B) **Immunohistochemical detection of ER, PR, and phospho-HER2 (Tyr877) in BT474 late stage lapatinib resistant cells. **(C) **Western blot analysis of UACC-812 and BT474 parental and resistant cell lines, including early (LR) and late (LLR) stage lapatinib resistant cells. Whole-cell extracts were analyzed by Western blot with the indicated antibodies. **(D) **BT474 parental, early, late stage lapatinib resistant, and combination (L + T) resistant cells were treated with fulvestrant for 24, 48, or 72 h and whole-cell extracts were analyzed by Western blot with the indicated antibodies.

The effect of F on resistant cell growth became apparent after Day 3 of the treatment (Figure 4A). To further assess the mechanism by which F inhibits the growth of the derivatives resistant to L-containing regimens, we treated parental, LR, and LTR UACC-812 and BT474 cells with F for 24, 48 and 72 hours, and probed for levels of ER-regulated gene expression and apoptosis molecules (Figures 4B and 5D). ER has been shown to activate genes associated with proliferation (for example, Cyclin D1) and with anti-apoptosis (for example, Bcl2 and survivin) in breast cancer cells [11,38]. In our study immunoblot analysis revealed that F induced degradation of ER in UACC-812 and BT474 derivatives after 24 hours of treatment. This led to down-regulation of Cyclin D1 and survivin in UACC-812 parental, LR, and LTR, but no induction of the apoptotic marker cleaved PARP was observed in parental UACC-812. In contrast, Bcl2 expression levels were increased in UACC-812 LR and LTR cells. This induced Bcl2 expression was inhibited in the presence of F and this was associated with induction of cleaved PARP in these cells. In BT474 LR and LTR no expression of Bcl2 and no significant down-regulation of Cyclin D1 was observed (Figure 5D). The proapoptotic Bcl2 family member Bik is down-regulated by estrogen [38] and, indeed, increased Bik and consequently cleaved PARP were observed in BT474 parental, LR, and LTR derivatives treated with F after 24 hours (Figure 5D). The magnitude of F-induced apoptosis, however, was probably greater in the resistant cells, based on the growth curve studies (Figure 4A). Interestingly, we did not observe an increase in AXL expression (data not shown), as previously described [20]. No inhibition of AKT activity was observed when BT474 LR or LTR were treated with F (Figure 5D). These results suggest that F through its antagonism of ER can overcome resistance to L-containing regimens, at least partly by regulating expression of Bik.

### The combination of endocrine and HER2-targeted therapy leads to strong inhibition of tumor growth and complete tumor regression in UACC-812 xenografts

**Figure 6:**
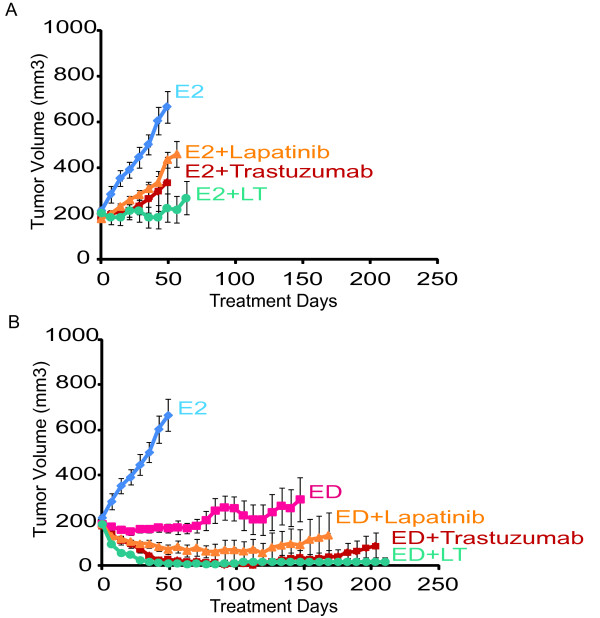
**Growth of UACC-812 xenografts treated with various anti-HER2 treatments, with or without estrogen deprivation**. **(A) **Treatment in the presence of estrogen supplementation, representing no endocrine therapy. Treatments included: Estrogen alone (E2) or with lapatinib (E2 + L), trastuzumab (E2 + T), or their combination (E2 + L + T). **(B) **Treatments in the presence of endocrine therapy in the form of estrogen deprivation. Treatments included: Estrogen (E2), estrogen deprivation (ED) alone, or along with lapatinib (ED + L), trastuzumab (ED + T), or their combination (ED + L + T). Results are presented as the mean tumor volume; error bars represent the standard error.

To further investigate if crosstalk between ER and HER2 is a mechanism of resistance to HER2-targeted therapy *in vivo*, using UACC-812 xenografts we compared the efficacy of the anti-HER2 regimens alone (Figure 6A) to block tumor growth versus their efficacy in combination with estrogen deprivation (ED) to also inhibit the ER pathway (Figure 6B). Anti-HER2 therapy alone (in the presence of estrogen) (E2) was only partially effective in slowing tumor growth and it did not lead to tumor regression (Figure 6A), though the combination of L plus T was superior to either monotherapy alone. The combination of ED together with anti-HER2 therapy was more effective than ED alone (Figure 6B). The more potent L + T combination together with ED achieved complete tumor regression in all mice with no recurrence after 210 days. Multiple comparisons between progression-free survivals (PFS) show that xenografts treated with the combination of endocrine and anti-HER2 therapy exhibit better response than with either anti-HER2 therapy alone (Additional files 3 and 4). Groups treated with ED plus L or ED plus the combination regimen displayed significantly improved PFS compared with the ED group. The combination of ED with various HER2-targeted treatments also exhibited better PFS than anti-HER2 therapy alone. These results suggest that simultaneous endocrine therapy together with an anti-HER2 drug combination like L + T is the most effective therapeutic regimen in this cell line.

### Switch from dependence on ER back to HER pathway dependence after prolonged, continuous L therapy in BT474 cells

When lapatinib-resistant BT474 cells were cultured for a longer period in the presence of lapatinib (more than six months) (BT474 Late LR, LLR), they acquired a more rapid, aggressive proliferative rate compared with BT474 LR at the early phase (46-fold versus 8-fold change of Abs 655 nm over nine days, Figure 5A). Down-regulated ER and PR expression and up-regulated phospho-HER2 were also observed in BT474 LLR cells (Figure 5B). Levels of phosphorylated EGFR, HER2, and HER3 increased in BT474 LLR as detected by immunoblot, compared to LR cells. There was no change in the levels of these proteins in long term lapatinib-treated UACC-812 LLR cells (Figure 5C). The increased protein levels of ER and PR observed in BT474 LR cells decreased in BT474 LLR, as the HER pathway became active once more. Furthermore, the extreme sensitivity to F was lost in the LLR cells, and there was no induction of Bik or evidence of apoptosis in BT474 LLR treated with F (Figure 5A and 5D). These results indicate that in some HER2-overexpressing breast cancer cells treated with L, ER can initially function as an escape pathway to cause resistant growth, only to be followed after more prolonged treatment by reactivation of the HER pathway, which once again becomes the driver of cell growth.

### Increased expression of HER2, HER3 and HER ligands accompany BT474 LLR growth

**Figure 7:**
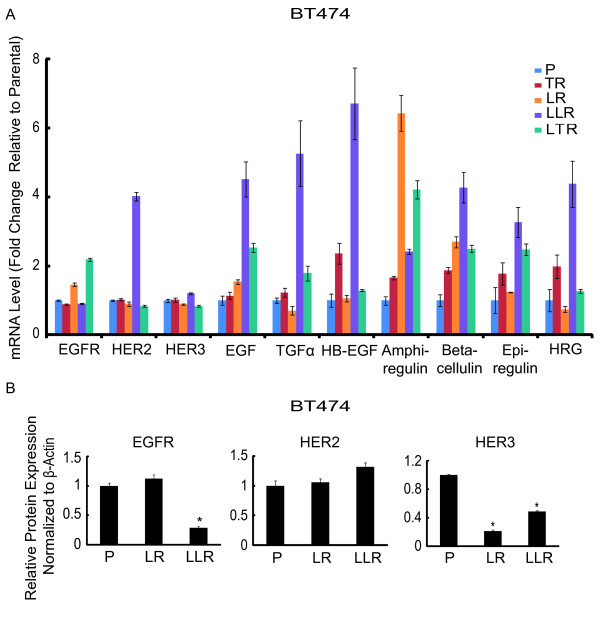
**BT474 late stage lapatinib-resistant cells overexpress HER2 and HER ligands**. **(A) **mRNA expression levels of HER receptors and ligands in BT474 parental and distinct resistant derivatives by qRT-PCR. Data were normalized to parental cells. **(B) **EGFR, HER2, and HER3 protein levels in BT474 parental, early, and late stage lapatinib-resistant cells. Protein level was quantified with Odyssey software (LI-COR Biosciences, Inc., Lincoln, NE). Each expression level was acquired from three independent samples for each derivative. Significance between groups was determined by multiple comparisons using the Sidak method (*P < 0.0001, EGFR expression: P versus LLR; HER3 expression: P versus LR, P versus LLR, or LR versus LLR).

Since BT474 LLR cells exhibited reactivated HER signaling activity, we also measured HER ligands and receptors in BT474 parental and resistant derivatives by qRT-PCR (Figure 7A). EGFR was up-regulated in BT474 LR and LTR, yet not in other derivatives. In contrast, expression levels of HER2, HER3, EGF, TGFα, heparin-binding EGF, betacellulin, and heregulin mRNA were all markedly increased in BT474 LLR compared with parental cells. Amphiregulin, which has been shown to be regulated by classical estrogen transcriptional activity, was noticeably increased in BT474 LR and LTR, but not in LLR in which ER signaling was once again low.

We then asked whether parental and LR BT474 derivatives expressed variable levels of HER receptor proteins (Figure 7B). BT474 LLR cells expressed decreased EGFR (*P *< 0.0001, P versus LLR) and HER3 (*P *< 0.0001, P versus LLR), but increased levels of HER2 (*P *= 0.0652, P versus LLR), while BT474 LR showed similar levels of EGFR and HER2 but down-regulated HER3 (*P *< 0.0001, P versus LR). BT474 LLR expressed higher levels of HER3 compared with the LR derivative (*P *< 0.0001, LR versus LLR). These data show that the transition of LR cells (driven by ER) to LLR (driven by HER signaling) is associated with increased levels of HER2, HER3, and several HER ligands.

**Figure 8:**
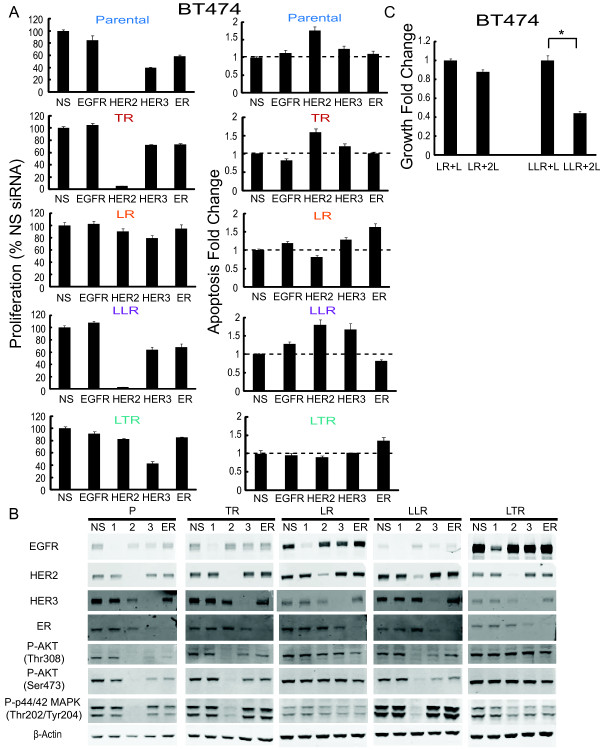
**Inhibition of HER2 restores lapatinib sensitivity in BT474 late stage lapatinib resistant cells**. **(A) **BT474 parental and resistant cells were treated with pooled EGFR, HER2, HER3, ER siRNA, or non-targeting control siRNA, for 72 hours. Proliferation was measured using the Click-iT EdU (5-ethynyl-2'- deoxyuridine) Microplate Assay. Apoptosis was measured by detecting Annexin V expression. Signals were visualized and quantitated by the Celigo cytometer (Cyntellect, San Diego, CA, USA). **(B) **Down-regulation of EGFR, HER2, HER3, and ER in BT474 derivatives after siRNA treatment was detected by Western blot. Whole-cell extracts were analyzed with the indicated antibodies, including downstream signaling. **(C) **Growth fold change of double dosage (2 μM) lapatinib on BT474 early and late stage-lapatinib resistant cells for six-day treatment. Cell numbers were assessed using methylene blue and quantified by absorbance at 655 nm and normalized against Day 0. Significance between groups was determined by multiple comparisons using the Sidak method (*P < 0.0001, LLR + L versus LLR + 2L).

To further assess the differential roles of the HER receptors and ER in BT474 parental and resistant derivatives, EGFR, HER2, HER3, and ER were depleted individually using small-interfering RNA (siRNA), and the effects on proliferation, apoptosis, and signaling were examined (Figure 8A, B, and Additional file 5). Parental BT474 were extremely sensitive to HER2 knockdown, which inhibited proliferation by 98%, induced apoptosis by 1.8-fold, and down-regulated expression of phosphorylated AKT and p44/42-MAPK. Although HER3 and ER siRNA suppressed the proliferation of parental BT474 more than 40%, no significant effects on apoptosis were observed. Like parental BT474 cells, the TR derivative was also extremely sensitive to HER2 siRNA, but less responsive to HER3 knockdown (only 28% inhibition of proliferation compared with 61% in parental cells). These results suggest that both parental and TR BT474 cells are highly dependent on HER2. Interestingly, knockdown of HER receptors and ER had little or no effect on the proliferation of BT474 LR and BT474 LTR, with the exception of HER3 siRNA, which inhibited the proliferation of BT474 LTR by 60%. However, ER siRNA induced a 1.6-fold increase in apoptosis in BT474 LR cells and a 1.4-fold increase in apoptosis in BT474 LTR cells, while siRNAs against all HER receptors caused little or no increase in apoptosis. These results are consistent with our previous findings, demonstrating induction of apoptosis by F but only a minimal effect on proliferation in both BT474 LR and LTR cells. In addition, the data also further implicate ER activity as an alternative survival pathway in BT474 LR and LTR cells.

In contrast, BT474 LLR cells showed extreme sensitivity to HER2 knockdown (97% inhibition of proliferation, 1.8-fold increase in apoptosis) and HER3 knockdown (1.67-fold increase in apoptosis). Levels of phospho AKT and p44/42 MAPK were inhibited in BT474 LLR cells subjected to HER2 siRNA. Furthermore, a double dosage of lapatinib (2 μM) suppressed BT474 LLR growth by 60% (*P *< 0.0001, LLR + L versus LLR + 2L), but had no major effect on BT474 LR (Figure 8C). Together with the results of the HER receptor quantitation (Figure 7A, B), these findings indicate that ER activity provides a survival stimulus for LR BT474 cells in the early phase of their acquired resistance; however, with more prolonged L treatment, levels of HER2, HER3, and multiple HER ligands increase, and the HER pathway once again becomes the dominant driver of proliferation and survival.

## Discussion

In this report we show that a dynamic transition between HER2 and ER activity plays a role in resistance to L-containing regimens, while sustained HER pathway activity is a prominent feature in TR cells. Our data suggest that ER-positive/HER2-positive cells, in general, exploit ER activity as a mechanism of *de novo *or acquired resistance to effective L-containing HER2-targeted regimens.

Four out of five ER-positive/HER2-positive cell lines in our panel showed up-regulation of ER signaling following treatment with combined L + T. However, only the MDA- MB-361 cell line, which showed the highest increase in ER activity upon L + T treatment, displayed a *de novo *resistance phenotype. Therefore, ER in this particular cell line acts as the dominant and primary driver of growth even before anti-HER2 therapy is initiated. The other ER-positive lines were initially sensitive to L + T treatment, but later ER was used as an escape pathway to cause acquired resistance to L + T. Thus, in ER-positive/HER2-positive breast cancer cells, either ER or HER2 can function initially as the major promoter of proliferation and survival. Eventually, however, with sustained, effective HER2 inhibition with L or L + T in these cell lines, ER becomes the primary driver of cell survival resulting in resistance to L or L + T therapy. These findings are consistent with two recent neoadjuvant trials in HER2-positive patients, where chemotherapy was administered in addition to HER2-targeted therapy. These trials demonstrated significantly lower pathological complete response rates (pCR) in ER-positive/HER2-positive than in ER-negative/HER2-positive tumors [39,40]. However, neither of these trials included ER-targeted therapy. One of these trials, which combined T plus the HER2 dimerization inhibitor pertuzumab [40], also included a group without chemotherapy. In this group, a 6% pCR rate was reported for the ER-positive tumors. A further recently reported neoadjuvant trial in patients with HER2-positive tumors, used L + T without chemotherapy but with combined endocrine therapy if the tumors were ER-positive [41]. This trial, which included patients with larger tumors, reported a 21% pCR rate, a pCR greater than three times that reported in the trastuzumab plus pertuzumab trial. Although it is difficult to compare across trials, the lower response rate in the T plus pertuzumab trial could be due to the failure of this regimen to target EGFR, ER, or both. Collectively, these results suggest that targeting the ER and HER2 pathways simultaneously in ER-positive/HER2-positive tumors is essential for obtaining optimal benefit. The results from our UACC-812 xenograft model, together with our previous findings in the MCF7-HER2 and BT474 models [24,31], demonstrate the capability and superiority of the potent L + T regimen in combination with endocrine therapy in achieving complete tumor regression and preventing the onset of therapeutic resistance. Therefore, these data strongly suggest a potential role for this strategy in the clinic.

Unlike UACC-812 LR and LTR, which exhibit no HER pathway activity, BT474 LR (early stage) and LTR maintain AKT activity, even in the presence of reduced HER receptor activity. Previously, sustained PI3K/AKT activity in BT474 LR clones was suggested to be regulated by AXL, a membrane-bound receptor tyrosine kinase [20]. In addition, ER has the ability to induce the expression of AXL, which could subsequently lead to activated AKT [20]. However, in our early BT474 LR derivatives, AXL expression was unchanged. When treated with F, BT474 LR displayed evidence of ER degradation, but no substantial effect on AKT activity was observed. These results suggest that other unknown mechanisms may also be maintaining PI3K/AKT activity in these cells.

While ER activity was dominant in the LR and LTR derivates of our cultured models, we found that HER2 activity was crucial for resistance to T, as siRNA knocking down HER2 in our TR derivatives inhibited proliferation and also induced apoptosis. One of the mechanisms of action of T is to disrupt ligand-independent HER2-HER3 heterodimer signaling [37]. UACC-812 and BT474 TR cells maintained high levels of EGFR and HER2 but showed decreased phosphorylated HER3, suggesting that T still manages to effectively disrupt HER2-HER3 heterodimer signaling in the resistant derivatives. Although it has been reported that EGFR and HER3 contribute to TR [19,42], our data demonstrate that HER2 is still required for growth in TR cells, while knockdown of EGFR or HER3 failed to elicit significant growth inhibition in BT474 TR. Importantly, the contribution of changes in antibody-dependent cell-mediated cytotoxicity (ADCC), thought to be one partial mechanism of action of T [43], could not be studied in our *in vitro *models. Therefore, in our culture studies, the observed inhibitory effect of T in comparison to L-containing regimens is related to the potency of this treatment directly on the HER signaling pathway. Collectively, we did show that TR derivatives are still dependent on the HER pathway and, therefore, remain sensitive to L, as previously reported [44].

Of note, we did not observe up-regulation of ER expression or signaling in the LR and LTR derivatives of HER2-positive/ER-negative cell lines, in which the HER2 pathway remains suppressed. However, further investigation, both *in vitro *and in the clinical setting, is required to evaluate whether more prolonged exposure to these HER2-targeted therapies will reactivate the ER pathway.

We found that HER3 expression levels increased upon commencement of HER2-targeted therapy, while HER2 phosphorylation was suppressed in most of our HER2-overexpressing models. Previous studies have indicated that AKT inhibition induces HER3 expression in HER2-positive cell lines [45], and consistent with this, AKT activity is significantly inhibited by HER2-targeted therapy in the majority of the models examined. SKBR3 and SUM-190 cells, however, maintain AKT phosphorylation and still up-regulate HER3 expression, suggesting that additional mechanisms must also control HER3 expression.

Reactivated HER signaling did confer resistance to L in BT474 cells but only after the cells had experienced a period of ER dependency. In contrast, UACC-812 LR cells were driven by ER activity and maintained a fairly stable phenotype even after prolonged L treatment. In BT474 LR cells, however, a switch in dependence from the ER to the HER2 pathway was observed during the late phase of acquisition of LR. In this model, enhanced ER activity reduced cell death in LR cells at the early stage, acting as a transitional pathway. Following prolonged treatment with L, a significant compensatory rearrangement of HER receptor and ligand expression occurred, ultimately leading to up-regulated levels of HER2, HER3, and many HER ligands. Interestingly, doubling the dose of L inhibited the HER2-dependent BT474 LLR cells, but not the ER-dependent BT474 LR cells. A therapeutic strategy that applies high doses of L intermittently has been shown to more effectively inhibit tumor growth in mouse models with minimal toxicity [46], a strategy that might be considered in the clinical setting. Another recent report suggests that up-regulated HER3 compensates for inhibition of L [18]. Although HER3 knockdown has no effect on BT474 early stage LR, HER3 siRNA induced increased apoptosis in BT474 LLR, suggesting that HER3 could contribute to LR. Repeat biopsy of tumors from patients with LR tumors might be helpful in differentiating those tumors with a greater dependence on ER from those that remain dependent on the HER pathway, thus acting as a guide to further therapy.

## Conclusions

The complexity and redundancy of the HER network requires more complete inhibition of the HER family of receptors with combination therapy. In cultured cells, treatment with L is more effective than T in achieving this inhibition, and the additive effect of the L + T combination achieves a more powerful blockade of the pathway than either therapy in isolation. In this study, we illustrate that TR derivatives show reactivation of the HER pathway as a mechanism of resistance. However, with a more complete HER2 blockade, resistance to L-containing regimens requires the activation of an alternative cell survival pathway. This is evident in ER-positive/HER2-positive cell lines, where up-regulation of the ER pathway occurs in order to create an escape survival pathway.

The findings of this study have several therapeutic implications: (i) A more potent HER pathway inhibitor, or a combination therapeutic strategy such as L + T, could improve the outcome of patients with HER2-positive breast cancer. Recent reports of clinical studies using L + T regimens support this idea. (ii) A combination of endocrine and anti-HER2 therapies given simultaneously might benefit ER-positive/HER2-positive patients, including those with tumors with low ER levels that clinically might be reported as ER-negative, especially if PR is still expressed. These ideas are currently being tested in clinical trials.

## Abbreviations

ADCC: antibody-dependent cell-mediated cytotoxicity; Cav-1: caveolin-1; E2: estrogen; ED: estrogen deprivation; EdU: 5-ethynyl-2'-deoxyuridine; EGFR: epidermal growth factor receptor; ER: estrogen receptor; F: fulvestrant; HER2: human epidermal growth factor receptor 2; IGF1R: insulin-like growth factor-I receptor; IHC: immunohistochemistry; L: lapatinib; LLR: late lapatinib resistant; MAPK: mitogen-activated protein kinase; P: parental; pCR: pathological complete response; PFS: progression-free survival; PI3K: phosphatidylinositol 3-kinase; PR: progesterone receptor; qRT-PCR: quantitative reverse transcription-polymerase chain reaction; R: resistant; RTK: receptor tyrosine kinase; SE: standard error; si-RNA: small-interfering RNA; T: trastuzumab; TKI: tyrosine kinase inhibitor

## Competing interests

RS and CKO have received research grant funding and payments for participating in advisory panels from GlaxoSmithKline and AstraZeneca. JG and GLP are employees of Genentech and hold shares of Roche.

## Authors' contributions

Y-CW contributed to concept design, established the resistant lines, carried out most of the molecular and functional studies, contributed to data analysis and interpretation, and drafted the manuscript. RS, CKO, and MFR contributed to concept design, data analysis and interpretation, and manuscript writing. GM, RG, RMW, XF, and MFB contributed to the establishment and characterization of the resistant lines and to the molecular and xenograft studies, and helped with data analysis. JG and GLP performed the qRT-PCR of the HER receptors and ligands and the data analysis. SGH contributed to concept design and performed the statistical analysis and data interpretation. GCC and NH contributed to data analysis and manuscript writing. All authors read and approved of the final manuscript.

## Supplementary Material

Additional file 1 **Primers and probes used in qRT-PCR experiments**. Click here for file

Additional file 2 **Characteristics of HER2-positive cell lines used in this study**. Click here for file

Additional file 3 **Pairwise comparison of progression-free survival (PFS) in the UACC-812 xenograft experiments by the Holmmel method**. Click here for file

Additional file 4 **Progression free survival (PFS) in UACC812 xenografts by Kaplan-Meier survival analysis**. Kaplan-Meier analyses illustrating PFS of xenografts treated with **(A) **estrogen (E2), estrogen deprivation (ED), E2 + trastuzumab (T), and ED + T; **(B) **E2, ED, E2 + lapatinib (L), and ED + L; **(C) **E2, ED, E2 + L + T, and ED + L + T. Click here for file

Additional file 5 **Representative fluorescent visualization of BT474 LLR treated with HER2 siRNA**. BT474 LLR cells were treated with pooled HER2 or non-targeting control siRNA for 72 hours, and then stained with Edu or Annexin V. The Edu, Annexin V, and DAPI stains are pseudocolored green, red, and blue respectively, and images were captured by the Celigo cytometer. Click here for file
