# Supplementary material for: Different mechanisms for resistance to trastuzumab versus lapatinib in HER2- positive breast cancers -- role of estrogen receptor and HER2 reactivation
Source: Breast Cancer Res. 2011 Nov 28;13(6):R121. doi: 10.1186/bcr3067 (PMC3326563; doi:10.1186/bcr3067)
Supplement: Additional file 1 — Primers and probes used in qRT-PCR experiments. [file bcr3067-S1.PDF]

Additional file 1: Primers and probes used in qRT-PCR experiments

ER:	5’-AACCGAGATGATGTAGCCAGC-3’ (F)
	5’-CAGGAACCAGGGAAAATGTG-3’(R)
PR:	5’-GATGCTGTATTTTGCACCTGATCTA-3’ (F)
	5’-GAACTCTTCT-TGGCTAACTTGAAGCT-3’(R)
actin:	5’-CC- CTGGCACCCAGCAC-3’ (F)
	5’-GCCGATCCACACGGAGTAC-3’ (R)
EGFR:	5’-GCCTTGAGTCATCTATTCAAGCAC-3’ (F)
	5’-TGCTACTGTCATTCGCACCTG-3’ (R)
	5’-FAM-AGCTCTGGCCACAACAG GGCATTTT-TAMRA-p-3’ (P)
HER2:	5’- TCTGGACGTGCCAGTGTGAA-3’ (F)
	5’-TGCTCCCTGAGGACACATCA-3’(R)
	5’-FAM-CAGAAGGCCAAGTCCGC- AGAAGCC-TAMRA-p-3’ (P)
HER3:	5’-GGGCAACTCTCAGGCAGTGT-3’ (F)
	5’-TGTACAGTGTCTGGTATTGGTTCTCA-3’(R)
	5’-FAM-CGCCGGTCACACT- CAGGCCAT-TAMRA-p-3’ (P)
EGF:	5’-GAGGACTGGCAAAGATAGAGTACG-T-3’ (F)
	5’-CTGGTTTTGCCAATGGATGA-3’(R)
	5-FAM-AGCATGCTGAAGC- CCTCATCACTGG-TAMRA-p-3’ (P)
TGF-α:	5’-GGACAGCACTGCCAGAGA-3’ (F)
	5’-CAGGTGATTACAGGCCAAGTAG-3’ (R)
	5’-FAM-CCTGGGTGTGCCA- CAGACCTTC-TAMRA-p-3’ (P)
HRG:	5’-TGGCTGACAGCAGGACTAAC-3’ (F)
	5’-CTGGCCTGGATTTCTTC-3’(R)
	5’-FAM-CAGCAGGCCGCTTCTCGACAC- TAMRA-p-3’ (P)

amphiregulin:	5’-ATATCACATTGGAGTCACTGCCCA-3’(F)
	5’-GGGTCCATTGTCTTATGATCCAC-3’(R)
	5’-FAM-AGCCATAAATGATGA-GTCGGTCCTCTTTCC-TAMRA-p-3’ (P)
HB-EGF:	5’-GAAAGACTTCCATCTA-GTCACAAAGA-3’ (F)
	5’-GGGAGGCCCAATCCTAGA-3’(R)
	5-FAM-TCCTTC- GTCCCCAGTTGCCG-TAMRA-p-3’ (P)
betacellulin:	5’-TGCCCCAAGCAATAC- AAGC-3’(F)
	5’-CGTCTGCTCGGCCACC-3’(R)
	5’-FAM-AAGCGGCATCTCCC-TTTGATGCAGTAA-TAMRA-p-3’ (P)
epiregulin:	5’-TGCATGCAATTTAAAGT-AACTTATTTGACTA-3’(F)
	5’-ATCTTAAGGTACACAATTATCAAAGCTGA-3’(R)
	5’-FAM-TCGGATTACTGAATTGTATCAATTTGTTTGTGTTCA-TAMRA-p-3’ (P)
HP1BP3:	5’-GCATTTGCTAGCTTTAGGTGCTT-3’ (F)
	5’-AGTTTAGGAAAGTCCAGGA-TTATTGC-3’(R)
	5’-FAM-TGGTGCCTGCCCTTTTCCTTGTTCA-TAMRA-p-3’ (P).

(F) = Forward Primer
(R) = Reverse Primer
P = TaqMan probe (FAM as reporter, TAMRA as quencher)
