# Supplementary material for: Different mechanisms for resistance to trastuzumab versus lapatinib in HER2- positive breast cancers -- role of estrogen receptor and HER2 reactivation
Source: Breast Cancer Res. 2011 Nov 28;13(6):R121. doi: 10.1186/bcr3067 (PMC3326563; doi:10.1186/bcr3067)
Supplement: Additional file 2 — Characteristics of HER2-positive cell lines used in this study. [file bcr3067-S2.PDF]

<u>Additional file 2</u> Characteristics of HER2-positive cell lines used in this study

Cell Line	Subtype	ER	PR	HER2	Mutation/overexpression
**AU565**	Luminal	-	-	Amp, oe	
**BT474**	Luminal	+	+	Amp, oe	TP53, PIK3CA
**HCC-1569**	Basal	-	-	Amp, oe	TP53 / Cyclin E
**HCC-1954**	Basal	-	-	Amp, oe	TP53, PIK3CA
**HCC-202**	Luminal	-	-	Amp, oe	PIK3CA
**MCF7-HER2**	Luminal	+	+	Amp, oe	
**MDA-MB-361**	Luminal	+	+	Amp, oe	PIK3CA
**MDA-MB-453**	Luminal	-	-	oe	PIK3CA
**SKBR3**	Luminal	-	-	Amp, oe	
**SUM-190**	Basal	-	-	Amp, oe	PIK3CA
**SUM-225**	Basal	-	-	oe	
**UACC-812**	Luminal	+	+	Amp, oe	PIK3CA
**ZR75-30**	Luminal	+	-	Amp, oe	

Information related to gene mutation and overexpression are summarized from [32, 35-36]. Amp, amplification; Oe, overexpression.
