# Supplementary material for: Different mechanisms for resistance to trastuzumab versus lapatinib in HER2- positive breast cancers -- role of estrogen receptor and HER2 reactivation
Source: Breast Cancer Res. 2011 Nov 28;13(6):R121. doi: 10.1186/bcr3067 (PMC3326563; doi:10.1186/bcr3067)
Supplement: Additional file 3 — Pairwise comparison of progression-free survival (PFS) in the UACC-812 xenograft experiments by the Holmmel method. [file bcr3067-S3.PDF]

<u>Additional file 3</u> Pairwise comparison of progression-free survival (PFS) in the UACC-812 xenograft experiments by the Holmmel method

	**E2**	**ED+T**	**ED+L**	**ED+LT**
**ED**	0.0002	0.2	0.001	0.001
**E2+T**	0.001	0.06		
**E2+L**	0.02		0.0004	
**E2+LT**	0.007			0.006
