# Supplementary figures and images for: Different mechanisms for resistance to trastuzumab versus lapatinib in HER2- positive breast cancers -- role of estrogen receptor and HER2 reactivation
Source: Breast Cancer Res. 2011 Nov 28;13(6):R121. doi: 10.1186/bcr3067 (PMC3326563; doi:10.1186/bcr3067)

**A**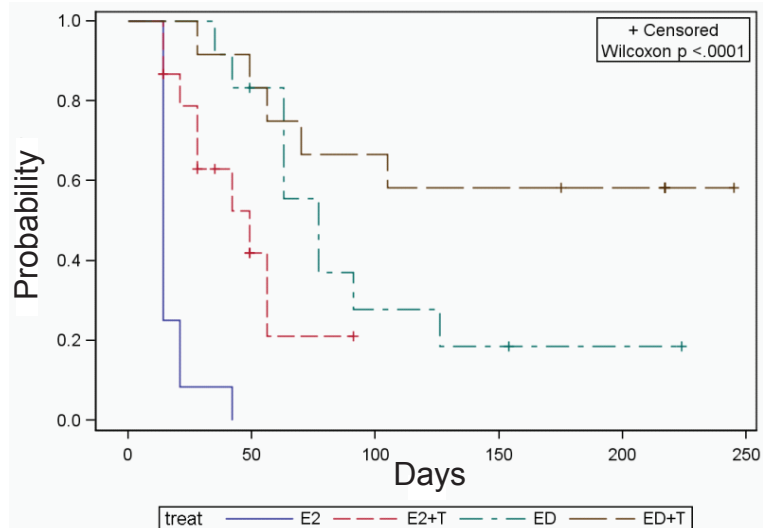**B**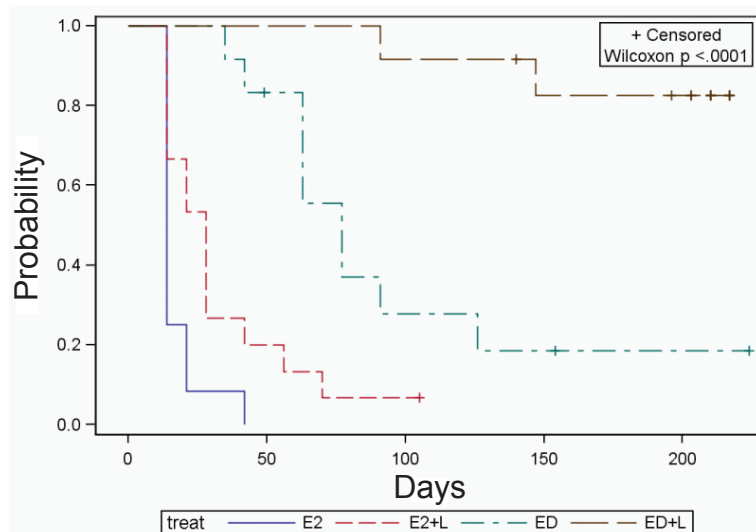**C**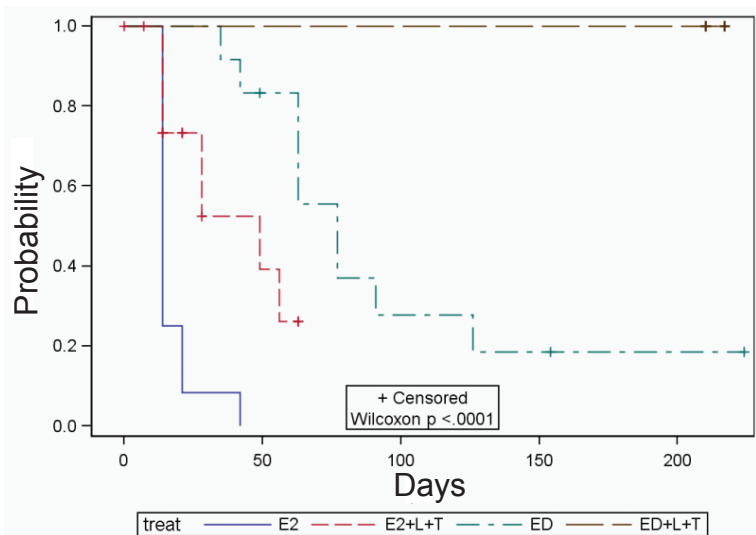

Supplement: Additional file 4 — Progression free survival (PFS) in UACC812 xenografts by Kaplan-Meier survival analysis. Kaplan-Meier analyses illustrating PFS of xenografts treated with (A) estrogen (E2), estrogen deprivation (ED), E2 + trastuzumab (T), and ED + T; (B) E2, ED, E2 + lapatinib (L), and ED + L; (C) E2, ED, E2 + L + T, and ED + L + T. [file bcr3067-S4.PDF]

# BT474-LLR

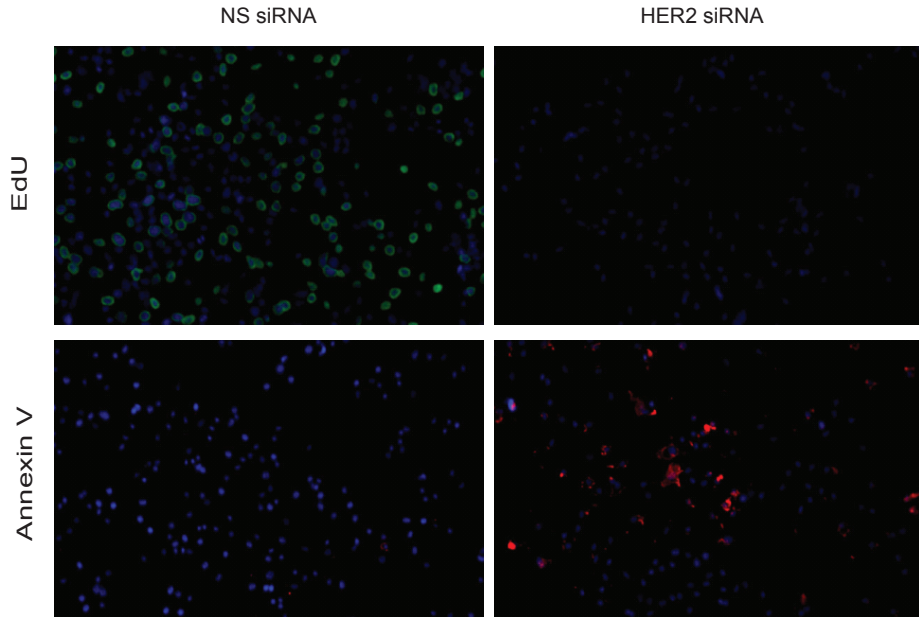

Supplement: Additional file 5 — Representative fluorescent visualization of BT474 LLR treated with HER2 siRNA. BT474 LLR cells were treated with pooled HER2 or non-targeting control siRNA for 72 hours, and then stained with Edu or Annexin V. The Edu, Annexin V, and DAPI stains are pseudocolored green, red, and blue respectively, and images were captured by the Celigo cytometer. [file bcr3067-S5.PDF]
